# Next generation drug clearance insights: real-time tracking in hepatobiliary and renal systems

**DOI:** 10.1038/s41377-025-01782-5

**Published:** 2025-02-26

**Authors:** Won Hur, Gi Hun Seong, Hak Soo Choi

**Affiliations:** 1 Gordon Center for Medical Imaging, Department of Radiology, Massachusetts General Hospital and Harvard Medical School, Boston, MA 02124 USA; 2 Department of Bionano Engineering, Center for Bionano Intelligence Education and Research, Hanyang University, Ansan, 426-791 South Korea

**Keywords:** Lasers, LEDs and light sources, Optical materials and structures

## Abstract

The integration of spatiotemporally resolved clearance pathway tracking (SRCPT) provides a new lens for evaluating drug clearance pathways, enabling precise mapping of physiological conditions of metabolic organs, such as liver or kidney impairment.

The efficient removal of drugs from the body, primarily through the liver and kidneys, plays a pivotal role in precision medicine. Understanding how drugs are cleared from the body with real-time imaging is vital for developing effective therapies. Traditionally, radioimaging and optical imaging have been used to track drug metabolism in living organisms, but these imaging modalities pose challenges such as radiation exposure or limited depth resolution, respectively. In a recent breakthrough, Lv et al. present a novel spatiotemporally resolved clearance pathway tracking (SRCPT) method^1^ based on photoacoustic tomography (PAT)^2^, offering noninvasive, real-time monitoring of drug clearance with relatively high spatial resolution. This innovation provides deeper insights into hepatobiliary vs. renal clearance pathways, which are key processes to understanding drug pharmacokinetics.

Renal and hepatobiliary systems represent the primary routes for drug elimination, each catering to different types of compounds. Figure 1 shows representative dual-channel imaging of renal clearance and hepatobiliary clearance using 700 nm emitting ZW700-1 and 800 nm emitting indocyanine green (ICG), respectively^3^. Renal clearance typically handles hydrophilic compounds or metabolites, eliminating them via glomerular filtration and tubular secretion^4–8^. The physicochemical properties of drugs, including the size, charge and polarity, composition, flexibility, mass-to-charge ratio, and hydrophilicity/lipophilicity, play a dominant role in this excretion pathway^9^. Thus, drugs with higher plasma protein binding tend to favor hepatic metabolism since they are less freely filtered through the kidneys. Nevertheless, physiological factors, including urine flow and renal blood flow, along with the unbound drug fraction and metabolites, also influence this process^10^. On the other hand, hepatobiliary clearance is largely responsible for metabolizing lipophilic drugs^11^ or highly charged drugs^12^ and excreting them into bile through liver enzymes such as cytochrome P450^13^. In addition to the physicochemical properties of injected drugs, variations in liver enzyme activity, blood flow, and plasma protein binding heavily influence this pathway, especially for drugs with a high hepatic extraction ratio^11^. This pathway can be affected by liver conditions such as cirrhosis or enzyme inhibition, leading to prolonged drug exposure and potential toxicity^14^. The choice of clearance pathway has significant clinical implications. For example, drugs cleared predominantly through the kidneys must be dose-adjusted in patients with renal impairment, as inadequate clearance can lead to accumulation and toxicity^15^.

**Fig. 1:**
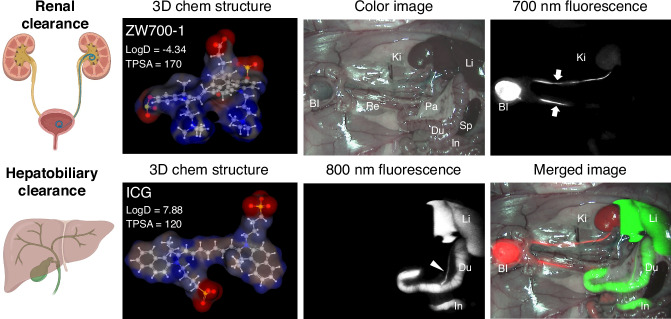
Representative images of renal vs. hepatobiliary clearance pathways. Dual-channel near-infrared (NIR) fluorescence imaging of ZW700-1 (700 nm) and indocyanine green (ICG; 800 nm) in the same animal. 50 nmol of each fluorophore was injected intravenously into a 250-g Sprague-Dawley rat 1 h prior to imaging. Bl bladder, Du duodenum, In intestine, Ki kidney, Li liver, Pa pancreas, Re rectum, Sp spleen. TPSA topological polar surface area. Arrows and an arrowhead indicate ureters and bile ducts, respectively. Images were created with Biorender. In silico calculations were calculated using MarvinSketch (ChemAxon, Budapest, Hungary). Adapted from ref. ^3^

Traditional imaging modalities, such as radiolabeling and optical imaging, have complemented each other, providing comprehensive real-time data on hepatobiliary and renal clearance. Radioimaging, including PET and SPECT, provides high sensitivity and quantitative 3D imaging of drug distribution and clearance pathways^16^. However, this modality requires radiotracers that may be costly and radioactive. Spatial resolution is typically limited, and repeated radioimaging leads to radiation exposure^17^.On the other hand, optical fluorescence imaging, such as near-infrared imaging (650–1700 nm), is non-ionizing and allows for real-time high-resolution imaging of drug clearance. However, this modality suffers from the limited penetration depth and phototoxicity risk. In addition, quantification can be less precise than radioimaging^18^.

Lv et al. in the recently published paper in *Light: Science & Applications*, leverage the unique capabilities of PAT to visualize drug clearance in real-time, overcoming the limitations of existing radiolabeling techniques^1^. SRCPT offers a non-ionizing alternative that penetrates deep tissues, achieving high-resolution, longitudinal monitoring of drug metabolism in organs, such as the liver and kidneys. This technique is enhanced by integrating a Monte Carlo-corrected empiric mathematical model, which addresses the challenge of laser fluence attenuation in deep tissues (Fig. 2). The result is a powerful imaging tool capable of dynamically mapping drug clearance with unprecedented accuracy. SRCPT was effectively used to track the clearance of mitoxantrone, a chemotherapeutic drug, in normal and liver-injured mice. The findings revealed that in mice with liver injury, hepatic metabolism of mitoxantrone was significantly reduced, leading to prolonged drug accumulation compared to healthy controls. This represents the power of SRCPT to detect subtle physiological changes in drug clearance pathways, offering vital insights for precision medicine. In addition, SRCPT showed its capability to distinguish various levels of renal dysfunction through monitoring immunoglobulin G (IgG) clearance patterns in acute kidney injury (AKI) models, highlighting its exceptional sensitivity in detecting organ-specific pathological conditions. Furthermore, Lv et al. validated the accuracy of the SRCPT technique by comparing it with PET imaging by using a double-labeled probe [68Ga]DFO-IRDye800CW, proving a strong correlation between SRCPT and PET^1^.

**Fig. 2:**
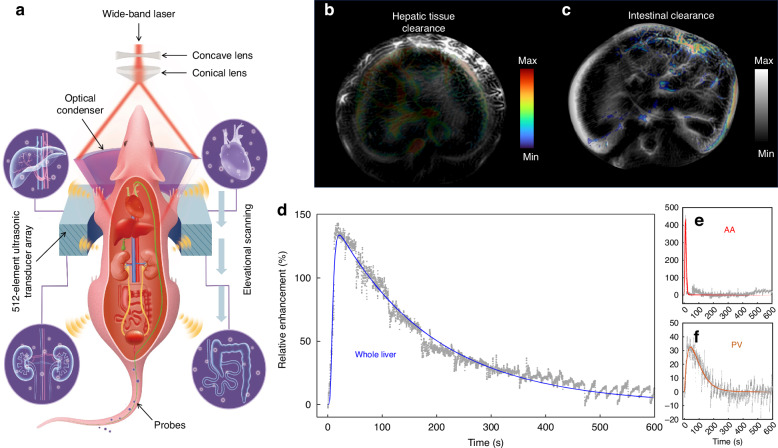
Spatiotemporally resolved clearance pathway tracking (SRCPT) method. **a** Schematic diagram of SRCPT of probes in the major organs. SRCPT of probe perfusion in the liver (**b**) and kidney sections (**c**), respectively. Percent relative enhancement of the quantitative PA signals generated by the injected dose in the whole liver (**d**), abdominal aorta (AA; **e**), and portal vein (PV; **f**), respectively. Adapted from ref. ^1^

The introduction of SRCPT opens new frontiers in drug clearance studies, particularly in distinguishing between renal and hepatobiliary clearance pathways. By providing real-time, detailed insights into how drugs are processed in different organs, SRCPT enables more informed decision-making in drug development, particularly when evaluating pharmacokinetics in disease models. For instance, drugs primarily cleared through the liver may require dose adjustments in patients with hepatic impairment, while renal clearance must be carefully monitored in patients with kidney disease^19^. Indeed, in AKI models, drugs accumulate in the kidneys without significant excretion, highlighting the impaired renal function.

The work by Lv et al. introduces a game-changing method for tracking drug clearance in vivo with SRCPT, providing unparalleled insights into the dynamics of hepatobiliary and renal drug elimination. The integration of advanced imaging algorithms further enhances its resolution and accuracy, establishing SRCPT as a critical tool for drug development and disease research (Fig. 2).

While SRCPT holds immense potential, there are still challenges to address. The accuracy of the method depends heavily on knowledge of optical properties in different tissues, and variations in these parameters can affect the precision of the Monte Carlo-based light fluence corrections. Additionally, the use of exogenous optical tracers in SRCPT means that certain drugs lacking sufficient absorption properties may not be suitable for this method^1^.

Future research should focus on expanding the range of compatible imaging tracers and improving the system’s adaptability to different drug types and physiological conditions. Despite these challenges, SRCPT represents a significant leap forward in our ability to study drug clearance and metabolism, offering new possibilities for precision medicine and personalized treatment approaches. By enabling real-time, high-resolution tracking of drug metabolism, SRCPT paves the way for more effective therapies tailored to individual patient needs, ultimately advancing the field of precision medicine.

## References

[1] Lv, J. et al. Dynamic synthetic-scanning photoacoustic tracking monitors hepatic and renal clearance pathway of exogeneous probes in vivo. *Light Sci. Appl.* **13**, 304 (2024). 39482292 10.1038/s41377-024-01644-6 PMC11528052

[2] Chen, R. H. et al. Photoacoustic molecular imaging-escorted adipose photodynamic-browning synergy for fighting obesity with virus-like complexes. *Nat. Nanotechnol.* **16**, 455–465 (2021). 33526836 10.1038/s41565-020-00844-6

[3] Hyun, H. et al. 700-nm Zwitterionic near-infrared fluorophores for dual-channel image-guided surgery. *Mol. Imaging Biol.* **18**, 52–61 (2016). 26084246 10.1007/s11307-015-0870-4 PMC4684479

[4] Choi, H. S. et al. Renal clearance of quantum dots. *Nat. Biotechnol.* **25**, 1165–1170 (2007). 17891134 10.1038/nbt1340 PMC2702539

[5] Yang, C. G. et al. ZW800‐PEG: a renal clearable Zwitterionic near‐infrared fluorophore for potential clinical translation. *Angew. Chem.* **133**, 13966–13971 (2021). 10.1002/anie.202102640 PMC8428668 33857346

[6] Ikeda, M. et al. Determination of renal function and injury using near-infrared fluorimetry in experimental cardiorenal syndrome. *Am. J. Physiol.-Ren. Physiol.* **312**, F629–F639 (2017). 10.1152/ajprenal.00573.2016 PMC5407071 28077373

[7] Kang, H. et al. Renal clearable nanochelators for iron overload therapy. *Nat. Commun.* **10**, 5134 (2019). 31723130 10.1038/s41467-019-13143-z PMC6853917

[8] Huang, Y. Y. et al. Physiological principles underlying the kidney targeting of renal nanomedicines. *Nat. Rev. Nephrol.* **20**, 354–370 (2024). 38409369 10.1038/s41581-024-00819-z PMC12875306

[9] Ahn, S. et al. Physicochemical descriptors in biodistribution and clearance of contrast agents. *Adv. Photon. Res.* **4**, 2300036 (2023). 10.1002/adpr.202300036 PMC12377636 40861064

[10] Du, B. J., Yu, M. X. & Zheng, J. Transport and interactions of nanoparticles in the kidneys. *Nat. Rev. Mater.* **3**, 358–374 (2018).

[11] Wasan, K. M. et al. Impact of lipoproteins on the biological activity and disposition of hydrophobic drugs: implications for drug discovery. *Nat. Rev. Drug Discov.* **7**, 84–99 (2008). 18079757 10.1038/nrd2353

[12] Owens, E. A. et al. Highly charged cyanine fluorophores for trafficking scaffold degradation. *Biomed. Mater.* **8**, 014109 (2013). 23353870 10.1088/1748-6041/8/1/014109 PMC3600611

[13] Ciută, A. D. et al. Structure of human drug transporters OATP1B1 and OATP1B3. *Nat. Commun.* **14**, 5774 (2023). 37723174 10.1038/s41467-023-41552-8 PMC10507018

[14] Arrese, M., Ananthananarayanan, M. & Suchy, F. J. Hepatobiliary transport: molecular mechanisms of development and cholestasis. *Pediatr. Res.* **44**, 141–147 (1998). 9702905 10.1203/00006450-199808000-00001

[15] Kellum, J. A. et al. Acute kidney injury. *Nat. Rev. Dis. Prim.* **7**, 52 (2021). 34267223 10.1038/s41572-021-00284-z

[16] Zhao, X. Y. et al. A rationally designed nuclei-targeting FAPI 04-based molecular probe with enhanced tumor uptake for PET/CT and fluorescence imaging. *Eur. J. Nucl. Med. Mol. Imaging* **51**, 1593–1604 (2024). 38512485 10.1007/s00259-024-06691-0

[17] Sajedi, S., Sabet, H. & Choi, H. S. Intraoperative biophotonic imaging systems for image-guided interventions. *Nanophotonics* **8**, 99–116 (2019). 31187017 10.1515/nanoph-2018-0134 PMC6559750

[18] Choi, H. S. & Kim, H. K. Multispectral image-guided surgery in patients. *Nat. Biomed. Eng.* **4**, 245–246 (2020). 32165731 10.1038/s41551-020-0536-7

[19] Anderson, G. D. & Hakimian, S. Pharmacokinetic of antiepileptic drugs in patients with hepatic or renal impairment. *Clin. Pharmacokinetics* **53**, 29–49 (2014). 10.1007/s40262-013-0107-0 24122696

